# Botulinum Toxin as a Pain Killer: Players and Actions in Antinociception

**DOI:** 10.3390/toxins7072435

**Published:** 2015-06-30

**Authors:** Dong-Wan Kim, Sun-Kyung Lee, Joohong Ahnn

**Affiliations:** 1 Department of Life Science, School of Natural Science, Hanyang University, Seoul 133-791, Korea; E-Mail: wizardbook@hanmail.net; 2 BK21 PLUS Life Science for BioDefense Research (BDR) Team, Hanyang University, Seoul 133-791, Korea; 3 The Research Institute for Natural Science, Hanyang University, Seoul 133-791, Korea

**Keywords:** botulinum neurotoxin, pain, nociception, neurotransmitter, neuropeptide, TRP

## Abstract

Botulinum neurotoxins (BoNTs) have been widely used to treat a variety of clinical ailments associated with pain. The inhibitory action of BoNTs on synaptic vesicle fusion blocks the releases of various pain-modulating neurotransmitters, including glutamate, substance P (SP), and calcitonin gene-related peptide (CGRP), as well as the addition of pain-sensing transmembrane receptors such as transient receptor potential (TRP) to neuronal plasma membrane. In addition, growing evidence suggests that the analgesic and anti-inflammatory effects of BoNTs are mediated through various molecular pathways. Recent studies have revealed that the detailed structural bases of BoNTs interact with their cellular receptors and SNAREs. In this review, we discuss the molecular and cellular mechanisms related to the efficacy of BoNTs in alleviating human pain and insights on engineering the toxins to extend therapeutic interventions related to nociception.

## 1. Introduction

Botulinum neurotoxins (BoNTs) are bacterial proteases produced by *Clostridium* *botulinum* and related species [1]. Infection by these bacteria results in a clinical condition called botulism. A hallmark of this potentially fatal disease is flaccid paralysis caused by interference with neurotransmitter release at presynaptic terminals. BoNTs (150 kDa) consist of a heavy chain (HC, 100 kDa) and a light chain (LC, 50 kDa) [2]. Of the 100 kDa, 50 kDa of the *C*-terminal region (H_C_) of the HC is a receptor-binding domain, which interacts with specific surface molecules of neurons. The other 50 kDa of the *N*-terminal domain (H_N_) is the translocation domain, which interacts with the active site of the LC, which is a metalloprotease. The seven serotypes of BoNTs, termed A–G, bind to and enter synaptic terminals and cleave one of the soluble *N*-ethylmaleimide-sensitive factor attachment protein receptor (SNARE) proteins, vesicle-associated membrane protein (VAMP), synaptosomal-associated protein 25 (SNAP25), or syntaxin [3]. These SNARE proteins mediate synaptic vesicle fusion; therefore, BoNTs inhibit the exocytosis of synaptic vesicles containing neurotransmitters [4]. Since Alan Scott treated strabismus by injecting BoNTs into extraocular muscles in the late 1970s, BoNTs have been widely applied in various therapeutic approaches associated with muscular, neurological, and secretory disorders because of their long-lasting high efficacy, patient tolerance, and satisfactory safety profile [5,6]. In addition to the list of the extensive therapeutic use of BoNTs, its use in reducing the clinical condition of pain has recently received attention. In this review, we will focus on the physiological, cellular, and molecular features of BoNTs as well as their mechanism related with action as anti-nociceptives.

## 2. BoNTs Complex: Molecular Machines Invading Epithelia and Nerves

### 2.1. The Components of BoNTs Complex

BoNTs are produced as a single inactive polypeptide, which is activated by a specific proteolysis on a surface-exposed loop subtended by a disulfide bond to generate an HC and LC [7]. H_C_ interacts with specific receptors such as certain gangliosides and synaptic vesicular proteins on neuronal cell surface. H_N_ serves as a translocating channel for LC, which is a zinc-containing metalloprotease harboring the typical His-Glu-X-X-His motif [8,9].

BoNTs holotoxins are assembled as non-covalently bound complexes of multiple protein components called progenitor toxin complexes (PTCs) [7,10]. These progenitor toxins contain several non-toxic neurotoxin-associated proteins (NAPs) such as three hemagglutinins (HA-17, HA-33, and HA-70) and a 140 kDa non-toxic non-hemagglutinin (NTNHA) protein. Clostridial bacteria produce 300 kDa of minimally functional PTC (M-PTC), which consists of BoNTs and NTNHA without HAs. Various combinations of HAs are added to assemble other PTCs, which range in sizes from 500 to 900 kDa, the largest PTC (L-PTC) [11]. These auxiliary proteins of HAs and NTNHAs function in stabilization, preservation and absorption of the BoNTs in intoxification process [7,12].

### 2.2. The Role of NTNHA

NTNHA interacts with BoNTs through a multivalent binding interface under acidic conditions (pH 6 or less), and the interlocking mode of interaction is dissolved at a neutral pH [13]. Electrostatic interactions play a major role in the pH-dependent binding and dissociation of BoNTs and NTNHA [7,13]. Recent site-directed mutagenesis studies based on structural analyses revealed that Glu982 and Asp1037 of BoNT/A are two key residues that mediate the pH-dependent binding between BoNT/A and NTNHA-A. These acidic amino acids are likely to be protonated at pH 6.0 during the BoNT/A-NTNHA interaction, leading to a stable M-PTC. However, they are likely to be deprotonated in a neutral or alkaline environment, thus generating repulsive charge interactions with NTNHA-A to destabilize M-PTC assembly. This induced fit between BoNT/A and NTNHA-A at an acidic pH is also evidenced by the solution structure of M-PTC as well as its crystal structure [14]. Thus, NTNHA shields BoNTs in the gastrointestinal tract under its low pH and protease-rich conditions and releases them upon entry into circulation during absorption from the intestine into the bloodstream.

### 2.3. HA and Gastrointestinal Absorption

While NTNHA shields BoNTs in the gastrointestinal environment, HAs facilitate the absorption of PTCs in the intestine because HA-containing PTCs are more efficient than free BoTNs in transepithelial transcytosis [15,16,17,18]. HAs bind to sugar moieties such as galactose and sialic acid on the apical surface of the intestinal epithelial monolayer [17,19]. Numerous biochemical evidences of HA-oligosaccharide interactions are also supported by X-ray crystallography of some HAs in PTC. HA-70b of BoNT/C has been reported to harbor a binding site for *N*-acetylneuraminic acid (Neu5Ac), and HA-33 of BoNT/C has been reported to have three binding sites that could interact with several sugars, including Neu5Ac, galactose (Gal), and *N*-acetylgalactosamine (GalNAc) [20,21,22,23].

**Figure 1:**
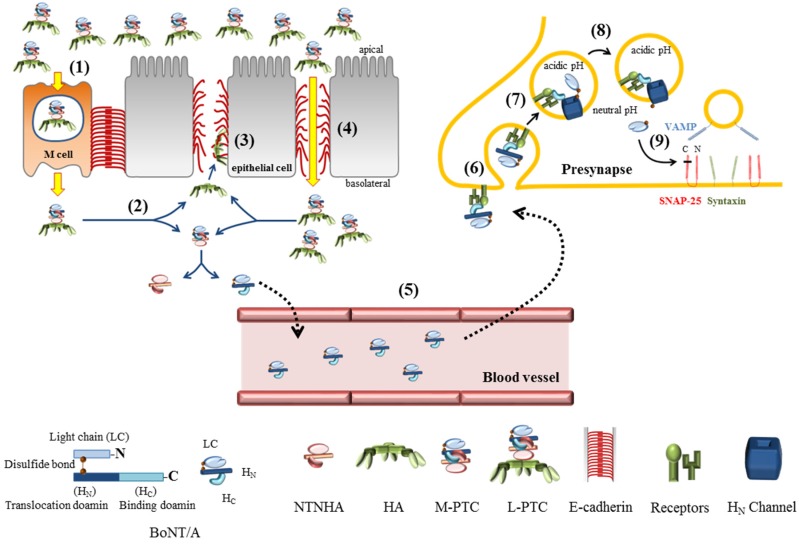
The route of Botulinum neurotoxin (BoNT)/A intoxication. (**1**) L-PTCs consisted of BoNT/A, non-toxic non-hemagglutinin (NTNHA), and HA are transcytosed through microfold (M) cells in human intestinal epithelia; (**2**) In the extracellular region where pH is neutral, progenitor toxin complexes (PTCs) are dissociated to release HA and M-PTC; (**3**) The trimeric HA complexes bind to E-cadherin, and rupture the adherence junctions in the intestinal epithelia; (**4**) More L-PTCs invade through the gap between the epithelial cells in the area in which the intestinal barriers are disrupted; (**5**) M-PTCs enter into the circulatory system and reach nerve terminals by an unknown way; (**6**) M-PTCs bind to their receptors such as polysialoganglioside (PSG) and synaptic vesicle glycoprotein 2 (SV2), and internalized via clathrin-dependent endocytosis; (**7**) H_N_ domains forms translocation channels for LC in an acidic environment inside endosomal vesicles; (**8**) LC is activated as a Zn^2+^-dependent protease in neutral pH in cytosol; (**9**) The LS protease cleaves SNAP-25 with extremely high specificity to block the exocytosis of synaptic vesicles.

BoNTs bind to polarized human intestinal epithelial cells and undergo transcytosis from the apical to the basolateral side [24]. It has recently been proposed that microfold (M) cells in the follicle-associated epithelium (FAE) of mouse Peyer’s patches (PPs) are the entry site of PTCs in the intestinal epithelium [25] (Figure 1). HA in the PTCs binds sugar moieties of glycoprotein 2 (GP2) on the M-cell surface, which does not have thick mucus layers. This binding requires the whole integrity of PTCs, because individual HA subcomponents or the HA2/HA3 core complex are not able to interact with GP2. Therefore, the interaction between the PTCs and GP2 on the surface of the M-cell is crucial for toxin’s absorption, because both M-cell depleted mice and GP2 knock-out mice are not susceptible to orally-administered PTCs. Thus, GP2 serves as a major endocytotic receptor for PTCs, which are translocated to the basolateral side of the epithelium, where PTCs are dissociated to release the free BoNT, NTNHA, and the HA complex.

The HA complex is a threefold symmetric heterododecameric complex, and as mentioned above, consisting of three HA70, three HA17, and six HA33 [19,26]. The trimeric HA binds to three E-cadherin (E-cad) ectodomains regardless of carbohydrate binding activity, which promotes the cell surface attachment of PTCs [12,19]. The recent phenomenal model based on the structure of the HA/A-E-cad crystallography suggests that the HA-E-cad interaction interferes with the trans-dimerization of E-cads in the adherens junctions on the cell surfaces between the intestinal epithelial cells. Thus, the intestinal barrier is disrupted, allowing more PTCs to invade the intestines via this paracellular route. Amino acid residues in the E-cad binding sites of HA are well conserved in the HAs of BoNT/A and BoNT/B, which mediate foodborne botulism in humans, but are barely conserved on the HAs of BoNT/C and BoNT/D, which predominantly cause botulism in birds and cattle [12]. Furthermore, the HAs of BoNT/A or B bind to human, bovine, or mouse E-cad with high affinity but not to chicken E-cad, whereas the HA of BoNT/C does not interact with human E-cad [27]. These observations help explain why BoNT/C and BoNT/D have hardly caused human foodborne botulism and why the oral consumption of BoNT/A rarely intoxicates avian species [3]. Although free BoNTs also bind intestinal epithelial cells, which also express the same receptors for BoNTs as neurons, only PTCs and none of the individual subcomponents of PTCs such as BoNTs, NTNHA, M-PTC, or HAs are able to compromise the integrity of the intestinal cell layers [12,28,29,30].

### 2.4. Uptake at Nerve Endings

After breaching the intestinal epithelial barrier and dissociating from PTCs, free BoNTs disperse in the extracellular fluid of the lamina propria inside the intestine and enter the lymphatic system and the blood circulation where they remain intact for several days [31]. How BoNTs cross the lymphatic and blood vessels is largely unknown, but BoNTs are unable to penetrate the blood–brain barrier and therefore cannot enter the central nervous system (CNS) via this route [32]. However, those robust BoNTs in circulation are hardly accumulated in most cells and organs; however, they primarily invade peripheral nerve terminals, most prominently at neuromuscular junctions [33,34]. The H_C_ domain of BoNTs is important in the recognition of nerve endings, and has a property to recognize multiple cell surface molecules [35,36,37]. The H_C_ initially binds to polysialoganglioside (PSG) with high affinity in the nM range [38]. Then, lateral mobility of PSG renders the anchored neurotoxins additional docking on members of the synaptic vesicle glycoprotein (SV) family [38,39,40,41,42]. The H_C_ of BoNTs generally binds to trisialoganglioside (GT)1b, disialoganglioside (GD)1b, and GD1a, which contain charged sialic acids [43,44]. The recent structural work on BoNT/A with a ganglioside analog has also confirmed the ganglioside-binding site [45]. BoNTs rapidly enter the synaptic vesicle lumen, mostly peripheral neurons *in vivo*, and the number of toxin molecules correlates with the number of SV2 molecules in the synaptic vesicle membrane [46,47,48]. The rate of entry for BoNT/A1 correlates with the rate of synaptic vesicle endocytosis, indicating that BoNTs exploit synaptic vesicle endocytosis to efficiently poison active synapses [39,49]. Although the mechanism of the entire endocytic pathway has not been completely defined yet, it has been reported that the uptake of BoNTs in neuronal cell lines relies on the clathrin-dependent pathway, and BoNTs are detected in early endosomal compartments [29]. Interestingly, the H_C_ of BoNT/A is homologous to fibroblast growth factors (FGFs), binds to FGF receptor 3 (FGFR3) in neuronal cells, and induces phosphorylation of the receptor, thus acting as an agonist [50]. In addition, both H_C_ and H_N_ are involved in the toxin binding process [51]. Therefore, the mechanism of toxin uptake in neurons possibly is more complicated than current models.

### 2.5. Translocation into the Cytosol to Be an Active Protease

To reach their target SNARE proteins in the cytosol of nerve cells, the catalytically active LC must be translocated from the endocytic vesicles into the cytosol. The belt region (residues 450–545) of H_N_ interacts with the active site of the LC at a neutral pH. Acidic pH in endosomes alters the conformation of H_N_ to allow efficient membrane interaction and membrane insertion of a series of elongated α-helices of H_N_, which form transmembrane channels with ion conduction properties [52,53]. Several different ion conductance states exist, reflecting discrete transient steps in the translocation of the LC and involve dynamic unfolding and refolding of LC after passage through the channels [54]. In this process, H_N_ also serves as a chaperone, preventing the aggregation of LC in the acidic environment of the endosomes, maintaining the unfolded conformation of LC during transit, and refolding LC in the neutral environment of the cytosol [55,56].

The disulfide bridge between H_N_ and LC that is maintained in the oxidizing environment of the endosomes must be reduced in the trans compartment of the cytosol to achieve efficient LC translocation [57]. The NADPH-thioredoxin reductase-thioredoxin redox system is mainly responsible for this disulfide reduction [58]. While the exact stoichiometry of BoNTs in the channels has not been clearly defined yet, the oligomeric channel model is supported by the AFM studies that detected the transition of monomeric BoNT/B at a neutral pH to a trimer upon ganglioside binding and acidification [59]. Although the H_C_ domain is not necessary for channel activity or LC translocation, its removal from the holotoxin releases the pH dependency of channel insertion into the membrane [60]. Therefore, channel formation and LC translocation are tightly associated in the structural context of BoNT multi-complexes, indicating that the components of the BoNT holotoxins work in concert as synchronized chaperones to achieve productive intoxication [55,61].

After its translocation into the cytosol from the endosomes in the presynaptic endings, the LC is refolded into a soluble Zn^2+^-dependent metalloprotease [42,62]. Various serotypes of the botulism neurotoxin cleave specific SNARE proteins. BoNT/A and E cleave SNAP-25. BoNT/B, D, F, and G; VAMP2; and BoNT/C hydrolyzes the integral plasma membrane protein syntaxin 1a and also SNAP-25 at higher concentrations of LC [9,63]. The completely assembled SNARE complex is resistant to cleavage; only free or loosely assembled SNARE proteins are susceptible to proteolysis by BoNTs [64]. The SNARE heterotrimeric complex plays a pivotal role in the fusion of the vesicular and plasma membrane lipid bilayers during the Ca^2+^-dependent fusion of synaptic vesicles containing neurotransmitters [65]. Therefore, the cleavage of SNAREs by BoNTs results in neuronal communication failure at synapses.

## 3. BoNTs: A Pain Killer

In addition to its well-known cosmetic uses, small doses of injected recombinant BoNT/A are widely used for clinical purposes to treat a variety of neuromuscular and autonomous disorders [66]. Since BoNT/A was approved by the U.S. Food and Drug Administration (FDA) in 1989 to treat strabismus, other clinical conditions such as hemifacial spasm, dystonia, spasticity, primary axillar hyperhidrosis, and urinary incontinence associated with an overactive detrusor muscle have been added to the list of approved uses [67]. Along with the expected neuromuscular effects, BoNT/A has been reported to reduce the pain associated with hyperactive muscular disorders [68]. Although decreased muscle contractions due to the inhibition of the release of acetylcholine at the neuromuscular junctions may indirectly contribute to pain relief, the low doses of BoNT/A necessary to affect pain relief, which often persists longer than the accompanying neuroparalytic effects, suggest the neurotoxin’s action on pain fibers and sensory, or autonomous nerves [69]. In addition to mitigating pain associated with hyperactive muscle contractions, the antinociceptive action of BoNT/A has been reported in various chronic pain associated with migraines and other types of neuropathic disorders [70,71]. Where and how BoNT/A acts in nociception are still largely in debate. The dominant opinion is that BoNT/A blocks the exocytosis of synaptic vesicles carrying neurotransmitters or inflammatory mediators, or that of other exocytic vesicles harboring pain sensors in peripheral sensory neurons. The hypothesis of BoNT/A’s central effects is highly controversial, due to the variability of experimental condition including the dosage of toxin treatment.

### 3.1. Antinociceptive Actions of BoNTs

#### 3.1.1. Glutamate, Substance P (SP) and Cacitonin-Gene Related Peptide (CGRP)

Inhibition of local neurotransmitter release by SNAP-25 cleavage, similar to the well-established intervention of acetylcholine release from neuromuscular end plates, has been suggested as one explanation of the neurotoxin’s antinociceptive effect [66,72]. The subcutaneous injection of BoNT/A into the hindpaws of rats prevented the nociceptive licking behavior induced by a formalin inflammatory challenge. This was accompanied by a reduction in formalin-evoked glutamate release without flaccid muscles [73]. A significant decrease of glutamate concentration was also reported in the human skin models of capsaicin-induced pain when pretreated with BoNT/A injections [74].

A number of studies using *in vitro* and *ex vivo* models have demonstrated the inhibition of the evoked release of proinflammatory neuropeptides. In rat models, BoNT/A inhibited the release of peripheral SP and CGRP in pain-induced bladders as well as in stimulated sensory neurons in culture [75,76,77,78].

#### 3.1.2. Transient Receptor Potential Vanilloids 1 (TRPV1)

Transient receptor potential vanilloids 1 (TRPV1) channels are activated by vanilloids such as capsaicin as well as by heat, protons, and various lipids, including endocannabinoids, anandamide, and *N*-arachidonoyl dopamine [79]. The multi-ligand sensing TRPV1 contributes to the detection of acute pain generated by heat and certain chemicals. The surface expression of TRPV1 in sensory neurons, particularly in association with nociceptive afferent fibers, is upregulated in some pathological conditions accompanied by elevated pain [80,81]. The local administration of BoNT/A decreased TRPV1 expressed in the suburothelial nerve fibers in the human bladder [82]. BoNT/A reduces the total expression of TRPV1 by inhibiting the exocytosis of TRPV1-harboring vesicles, which leads to the proteosomal degradation of TRPV1 [83,84]. Therefore, the potential therapeutic utility of BoNT/A has extended to the modulation of TRPV1 surface expression in pain-associated pathological conditions.

#### 3.1.3. GABAergic and Opioidergic Neurotransmission

While the mechanism of the central modulation of sensitization by BoNT/A has been largely unknown, several research approaches utilizing antagonists against neurotransmitter receptors have linked γ-aminobutyric acid (GABA) and opioid transmission to the central antinociceptive action of the toxin. Both GABAergic and opioids released by local circuit interneurons and inhibitory descending fibers in the spinal cord are able to strongly attenuate the sensory input transmitted to the dorsal horn [85]. The ionotropic GABA-A receptor antagonist bicuculline, injected both intraperitoneally and intrathetically, prevented the reduction of BoNT/A-mediated nocifensive behaviors such as licking, flinching, and shaking of a formalin-injected paw. In addition, these injections prevented the antiallodynic effect of the toxin in partial sciatic nerve transection-induced mechanical allodynia [86]. However, bicuculline injected into the cerebellomedullary cistern had no effect, suggesting that BoNT/A affects GABAergic transmission at the spinal cord and not at the supraspinal level. Similarly, both naloxozanine and naltrexone, selective and non-selective μ-opioid receptor antagonists, prevented the antinociceptive activity of BoNT/A in the formalin test and sciatica nerve transection neuropathy models [87]. These findings also aide in an understanding of previous studies that showed the synergistic activity of morphine and BoNT/A on inflammatory and neuropathic pain as well as the prevention of the development of morphine-induced tolerance by peripheral BoNT/A application [88,89].

#### 3.1.4. Central Effects of BoNTs in Rat Models

**Figure 2:**
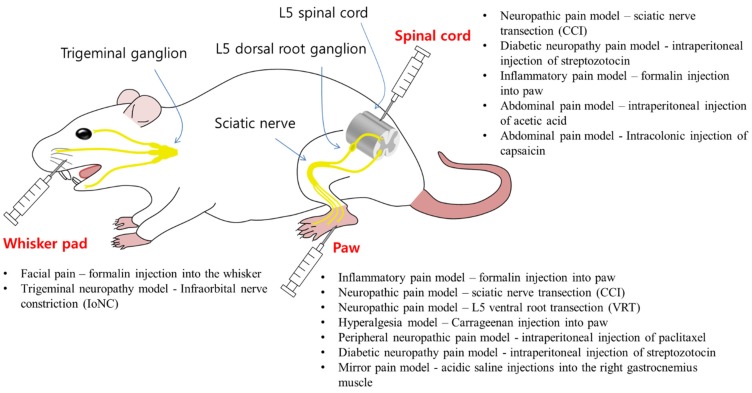
Pain Models of Rat. BoNT/A is peripherally injected into whisker pads or paws, or intrathetically into spinal cord of rats with indicated pain models. BoNT/A is suggested to move via axonal transport to reach central nervous system, and to inhibit the release of neurotransmitters in interneurons or descending fibers.

Although BoNT/A affects pain relief primarily at peripheral nerves in locally administered areas, the possibility of centrally mediated antinociceptive action has been proposed [66] (Figure 2 and Table 1). One piece of experimental evidence supporting CNS effects is the contralateral effect of BoNT/A at sites distant from the injection site in rats. Contralateral injection of several units of BoNT/A reduced hyperalgesia induced by intramuscular injection of acidic saline, which was sensitive to the microtubule depolymerizing drug colchicine, which was ipsilaterally injected into the sciatic nerves [90,91]. In another study, BoNT/A injected into one hindpaw reversed bilaterally decreased hindpaw mechanical withdrawal thresholds induced by chemotherapeutics [92]. The effects of BoNT/A on the distant contralateral side from the region of administration were also reported in rat models of neuropathic pain induced by ventral root transection, infraorbital nerve constriction, and trigeminal neuropathy [93,94,95]. Therefore, these studies reporting centrally mediated BoNT/A effects have led to the assumption that BoNT/A is transported from the site of injection.

#### 3.1.5. Axonal Transport of BoNTs

The possibility that BoNT/A could reach the CNS via retrograde transport mediated by microtubule tracks along axons was already suggested many years ago by researchers studying radiolabeled BoNT/A [96,97]. Later ultrastructural autoradiographic studies also showed that BoNT/A is retrogradely transported within the axonal compartment of peripherally injected regions [33]. However, because of the prevailing view that BoNT/A axonal transport is either too slow or very limited, it was argued that the toxin’s activity did not remain to affect the CNS. More recent research has demonstrated that BoNT/A may be retrogradely transported by central and peripheral neurons, and then transcytosed to post synaptic neurons where the toxin remains active [98]. In the study, after BoNT/A was injected into the central and peripheral regions, truncated SNAP-25 appeared not only at the injection site but also in distant regions projecting from the administered area. An unilateral injection of BoNT/A in the hippocampal region, the optic tectum, and the center of the whisker pad resulted in detectable SNAP-25 cleavage in the contralateral untreated hemisphere, in synaptic terminals within the retina, and in the facial nucleus, respectively. The retrograde movement of the toxin was inhibited by colchicine, indicating the involvement of microtubule-dependent axonal transport. Although the study shows the spread of toxins injected at the extremely high dose is restricted in the area which projects to the toxin-infusion region, it is unlikely that low dose of most clinical uses may cause similar effect. In another research, BoNT/A injected into the whisker pad area alleviated mechanical allodynia induced by infraorbital nerve constriction and reduced exaggerated neurotransmitter release from trigeminal ganglion neurons [99]. These studies suggest that peripherally injected BoNT/A moves along peripheral nerves via retrograde axonal transport, thus reaching central nerves in which a toxin protease inhibits neurotransmission by a SNAP-25 cleavage. While the retrograde transport may be responsible for the displacement of peripherally injected BoNT/A at the level of the spinal dorsal horn where it may inhibit the spinal release of neurotransmitters, it should be noted that antinociception was assayed mostly based on the behavioral response requiring motor neuron activity, which might have been affected by application of toxin.

The intracellular pathways underlying the proposed multiple inter-neuronal transport remain largely unclear. It has been observed that BoNT/A and BoNT/E internalized in spinal cord motor neurons were carried in non-acidic axonal trafficking organelles that are largely independent of the stimulated synaptic vesicle recycling process [32]. Some of those BoNT-containing vesicles also carried a well-known CNS targeting tetanus toxin, when added together with BoNTs, thus indicating that BoNTs are capable of taking advantage of the fast axonal retrograde transport compartment, which is composed of multifunctional trafficking organelles orchestrating the simultaneous transfer of diverse cargoes from nerve terminals to the soma. Recently, it has been shown that BoNT/A is capable of spreading not only reterogradely but also anterogradely in the CNS [100]. When injected into rat eyes, a cleaved SNAP-25 was detected in the superior colliculus (SC) and in presynaptic structures of the tectum but not at retinal terminals. These results indicate that catalytically active BoNT/A is anterogradely transported from the eye to the SC and transcytosed to tectal synapses. These studies used several orders of magnitude higher concentration of toxins than liberally-used clinical application. Above all, BoNTs were not detected at all in distant neurites, in case added to primary cultured neurons at the concentration comparable to widely-used clinical dosage [101]. Therefore, fast axonal transport may occur in neurons at injected region, but its proposed role as a general gateway to the CNS for the delivery of peripheral BoNTs should be rigorously examined.

**Table 1:** Antinociceptive effects of BoNTs in rat pain models.

BoNT/A injection	Pain model	Antinociceptive effects
Paws	Formalin induced inflammatory pain model [73,87,102]	Reduction of enhanced nocifensive behaviors (licking, flinching and shaking) [87,102]
		Reduction of c-fos early response gene expression [87,102]
		Reduction of enhanced glutamate release in primary afferent terminals [73]
	Sciatic nerve transection (CCI) induced neuropathic model [87,91,102,103,104]	Recovery of paw withdrawal response [87,91,102,103,104]
		Cleaved cSNAP-25 detected in paw, sciatic nerve, DRG, and L4/L5 spinal cord (dorsal horn) [103]
		Recovery of thermal hyperalgesia [91,104]
	L5 ventral root transection (VRT) induced neuropathic model [93,94]	Bilateral recovery of decreased paw withdrawal thresholds [93,94]
		Reduced expression of TRPV1 and P2X3 in dorsal root ganglion [93,94]
	Carrageenan-induced hyperalgesia [92,105]	Recovery of paw withdrawal response [92,105]
		Recovery of thermal hyperalgesia [105]
		Reduction of c-fos early response gene expression in spinal cord [105]
	Paclitaxel-induced peripheral neuropathy model [92]	Bilateral recovery of decreased paw withdrawal thresholds [92]
	Diabetic neuropathy pain model [106]	Bilateral recovery of decreased paw withdrawal thresholds [106]
		Bilateral recovery of mechanical and thermal hypersensitivity [106]
	Acidic saline induced pain model [90]	Bilateral recovery of decreased paw withdrawal thresholds [90]
Spinal Cord	Sciatic nerve transection (SCI) induced neuropathic model [104]	Reduction of mechanical allodynia and thermal hyperalgesia [104]
	Diabetic neuropathy pain model [106]	Bilateral recovery of decreased paw withdrawal thresholds [106]
		Bilateral recovery of mechanical and thermal hypersensitivity [106]
	Formalin induced inflammatory pain model [107]	Reduction of enhanced nocifensive behaviors (licking, flinching and shaking) [107]
		Reduction of CGRP in spinal dorsal horn [107]
	Acetic acid induced abdominal pain [86]	Reduced writhes [86]
		Reduction of increased c-fos expression in dorsal horn of the spinal cord (S2/S3segments) [86]
		Reduction of mechanical allodynia [86]
Face	Formalin-induced facial pain (into the whisker pad) [108]	Reduction of facial rubbing [108]
		Cleaved cSNAP-25 detected in trigeminal nucleus caudalis (TNC) [108]
		Colchicine-sensitive [108]
	Infraorbital nerve constriction (IoNC) induced trigeminal neuropathy model [95]	Reduction of dural extravasation [95]
		Colchicine-sensitive bilateral analgesic effect in trigeminal ganglion [95]

### 3.2. Molecular Therapeutics of BoNTs

Targeting specific pain-processing neurons expressing particular receptors is a potential new way of treating pain. The LC of BoNT/A conjugated with SP successfully targeted NK1 receptors in nociceptive neurons in the trigeminal nucleus caudalis in a murine model of Taxol-induced neuropathic pain, thus reducing thermal hyperalgesia [109]. P2X purinoreceptor 3 (P2X3), a purinoreceptor for ATP, is predominantly expressed on nociceptive sensory neurons and is critical in chronic inflammatory neuropathic pain [110]. Recent approaches using a single chain antibody (scFv) against the extracellular domain of P2X3 have shown that a fusion protein of scFv and an activated di-chain form of BoNT/A without the C-terminal binding subdomain inhibits the release of pain mediator peptides by cleaving SNAP-25 [111]. Therefore, the targeted delivery of a SNARE protease to specific sets of neurons may be an attractive therapeutic potential, and more trials with extensive and elaborate strategies aiming at different molecules are expected in the near future.

## 4. Conclusions

Recent collaborative research efforts with complementary expertise have been fruitful to elucidate the mechanics of BoNTs actions in GI track and neurons (Figure 1). Especially, the molecular architecture of multi-subunit toxin complexes, the genetically engineered mammalian models, and the electrophysiological approach with structure-based insights have provided an unprecedented wealth of knowledge of the structure and a function of BoNTs.

The 3D EM reconstruction of L-PTC of BoNT/A and the HA/A-E-cad crystallography have recently provided insightful information to support toxin’s incredible stability in a harsh condition in the gastrointestinal environment and the facilitated absorption mechanism in gastrointestinal epithelia [12,19]. The crystal structure of M-PTC of BoNT/A reveals the interlocking mode of binding between BoNT/A and NTNHA-A, which is pH sensitive so that induces a strong binding for protection in the gut and releases toxin to enter the circulatory tract [13]. Identifying GP2 on the M-cell surface in the intestinal epithelia as a receptor for HA in the PTCs is the breakthrough to understand the oral biologics of BoNTs [25]. M cell-depleted and GP2 deficient mice are markedly less susceptible to the toxicity driven by the food-born botulism model. The translocation of a LC protease across endosomes results from the orchestrated chaperoning activity of each functional domain of LC, TD and HC [52]. Subtle conformational change of minimum channel-forming truncation TD by acidification inside endosomes suggests larger structural rearrangement of LC unfolding tightly associated with TD channel formation.

The widely reported antinociceptive effect of BoNT/A was thought to be primarily mediated by the blocking of neurotransmitter and inflammatory substance release, and the inhibition of plasma membrane insertion of pain sensors at peripheral level. However, observations of bilateral action in distant region after unilateral injection of BoNT/A suggest the hypothesis that peripherally administered BoNT/A spreads out to central region via axonal transport to target neurotransmission of pain sensory circuits. Cleaved SNAP-25 was also detected in central areas, to which sensory and motor neurons in BoNT/A-injected peripheral regions are projected (Figure 2, Table 1). However, the currently known central effect of neurotoxins is very controversial, especially regarding the experimental condition such as the dosage of toxins. Further investigation about the central action of BoNT/A must be one of the next moves in the BoNT biologics research field, and will provide invaluable information helping understand pathophysiology of chronic pain, and for further development of BoNT/A use in pain and other clinical and cosmetic indications. At last, but not least, molecular engineering of BoNT fusion proteins with scFV or neuropeptides specific to surface receptors of certain neurons or cells awaits growing attention to be a potential new therapeutic intervention to treat various illness even beyond pain.
